# The Oxford hip score: the patient's perspective

**DOI:** 10.1186/1477-7525-3-66

**Published:** 2005-10-31

**Authors:** Vikki Wylde, Ian D Learmonth, Victoria J Cavendish

**Affiliations:** 1 Academic Orthopaedic Unit, University of Bristol, Avon Orthopaedic Centre, Southmead Hospital, Westbury-on-Trym, Bristol BS10 5NB UK

## Abstract

**Background:**

In the last 25 years, assessment of orthopaedic intervention has become patient focused, with the development of self-completion patient-centred outcome measures. The Oxford hip score (OHS) is a joint specific outcome measure tool designed to assess disability in patients undergoing total hip replacement (THR). Although the psychometric properties of the OHS have been rigorously examined, there is little research on the patient's perspective of the OHS. Therefore, the aim of this study is to assess whether the OHS is an adequate disability measure from the patient's perspective using qualitative analysis of annotations written on the OHS by patients.

**Methods:**

In total, 276 orthopaedic patients completed an OHS between April 2004 and May 2005. One hundred and fifty six pre-operative patients listed for a THR completed the OHS during a pre-admission assessment clinic, and 120 post-operative patients completed the OHS postally in the home setting. Patient's unprompted annotations in response to the questions on the OHS were recorded and grouped into thematic categories.

**Results:**

In total, 46 (17%) patients made 52 annotations when completing the OHS. These annotations identified five main areas of difficulty that patients experienced: lack of question clarity (particularly concerning the use of aids), difficulty in reporting measurements of pain, restrictive and irrelevant questions, the influence of co-morbidities on responses, and double-barrelled questions.

**Conclusion:**

Although the OHS is a useful short tool for the assessment of disability in patients undergoing THR, this study identified several problem areas that are applicable to patient-centred outcome tools in general. To overcome these current limitations, further work is underway to develop a more individualised patient-centred outcome measure of disability for use in patients with osteoarthritis.

## Background

During the last decade, the assessment of outcomes in orthopaedic surgery has shifted from the success or failure of an implant towards patient satisfaction and quality of life [1]. Initially, surgeon assessment of total hip replacement (THR) outcome was accepted, with the development of tools such as the Harris Hip Score [2] and the Charnley score [3]. However, these measures presuppose a concordance between the views of patients and clinicians, which has been proved to be an erroneous assumption [4,5], particularly in subjective domains such as pain [6]. Consequently, the last 25 years has witnessed the development of generic and disease-specific self-completion patient-centred outcome measures. Generic measures such as the SF-12 [7] and Nottingham Health Profile [8] endeavour to assess all important dimensions of health-related quality of life [9]. Disease-specific tools such as the Arthritis Impact Measurement Scale (AIMS) [10] and the Western Ontario and McMaster University Osteoarthritis Index (WOMAC) [11] focus on specific aspects of disability relating to a particular condition. These are supplemented by joint specific measures such as the Oxford hip score (OHS) [12] and the Hip Disability and Osteoarthritis Outcome Score (HOOS) [13].

The OHS is a patient-centred questionnaire that is designed to assess functional ability and pain from the patient's perspective. It is a short, twelve-item questionnaire developed for completion by patients undergoing THR [12] and is extensively referenced in the orthopaedic literature [14-21]. The OHS has been demonstrated to be highly sensitive to change in patients undergoing primary THR [12,16,21,19,22] and revision THR [15,16]. It correlates well with patient satisfaction [15,19] and other patient-centred instruments, such as the Euroqol 5D [15]. Responsiveness of the OHS to change has been found to be greater than generic measures such as the SF-36 [16,18] and disease specific measures such as the WOMAC [21]. The OHS has been utilized in a broad range of contexts, including studies comparing different prostheses [14], surgeon and patient expectations [20], and the outcomes of NHS and private patients [17].

Although the OHS has been shown to have internal consistency and produce data of high reliability and validity [12], there is a shortage of published data on the patient's perception of the OHS. During the validation of the questionnaire, there was no reference to difficulties that patients experienced when completing the OHS, beyond a brief statement that "the patients had little difficulty in completing it" [12]. Previous research has explored patient's perception of the OHS, and found that patients encountered several limitations of the OHS relating to question specificity, response category clarity, exclusion of comorbidities, and experience of pain [23]. However, this study was limited to a small sample size and during the past half decade no further work has been published investigating the patient's experience of the OHS. Therefore, the aim of this study was to determine, from the patient's perspective, if the OHS is an adequate questionnaire for measuring disability. This was achieved by analysing unprompted, spontaneous annotations generated by patients completing a paper copy of the OHS.

## Methods

Between April 2004 and May 2005 patients attending orthopaedic preadmission assessment clinic at the Avon Orthopaedic Centre, under the care of one consultant orthopaedic surgeon (IDL), and awaiting THR, were administered an OHS as part of a routine questionnaire pack used in the clinic. These patients were sampled as they were expected to be unfamiliar with the OHS, as the introduction of this questionnaire into routine clinical care in this clinic was initiated in April 2004. Between January 2005 and May 2005, consecutive patients with 12-months follow-up, who received a THR under the care of a consultant orthopaedic surgeon (IDL), completed a postally administered OHS as part of their on-going clinical care. In addition, all patients that had an IPS Stem (DePuy) between 1997 and 2004, under the care of one consultant orthopaedic surgeon (IDL) complete a postal OHS as part of another study. During administration the patients were not instructed to annotate or comment on the questions on the OHS.

**Figure 1:**
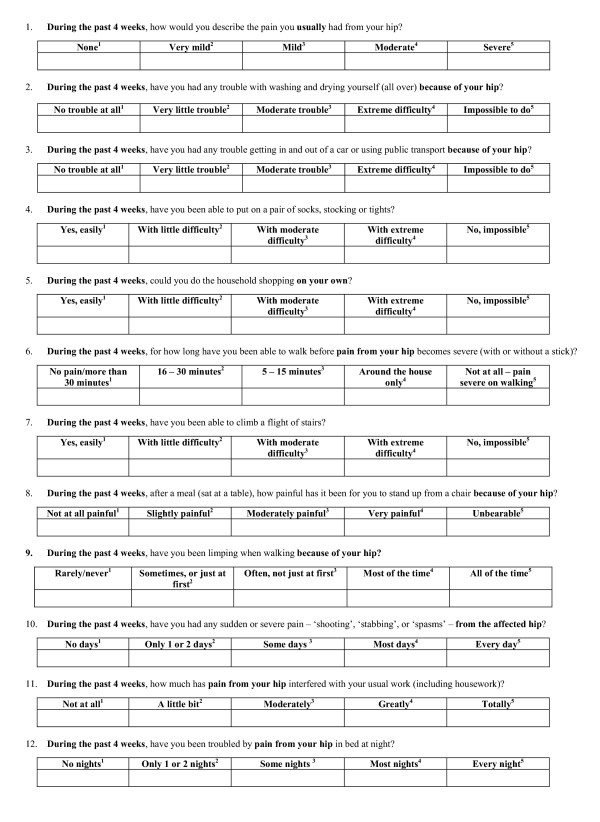
The format of the Oxford hip score.

The OHS consists of 12 questions about pain and disability experienced over the past four weeks. Each item has five response categories, given a score of between 1–5 (low disability to high disability). Scoring involves summating the total for each item to produce a final score between 12–60, with a higher score indicating greater disability. In this study the OHS was presented on a double-sided sheet of A4 paper, with six questions on each side. The response categories to each question were formatted as a Likert scale (Figure 1) with the coding frame integrated into the questionnaire. Both the questionnaires administered in the pre-admission clinic and the postal questionnaires were formatted in an identical manner.

Each questionnaire was reviewed for spontaneously generated annotations and these annotations were then grouped into thematic categories.

## Results

In total, 276 patients completed the OHS questionnaire. In pre-admission assessment clinic, 156 consecutive patients, listed for a THR, completed an OHS. For patients that attended the clinic twice between April 2004 and May 2005, only the first questionnaire was included in the analysis. Post-operatively, 120 patients completed a postally administered OHS. The pre-operative mean OHS was 44.1 (SD 8.3, range 21–59) and the mean post-operative OHS was 24.1 (SD 11.3, range 12–55). Patients who completed the OHS after surgery had a mean follow-up period of 24 months (SD 19, range 12–77 months). The sample consisted of 169 women (61%) and 107 men (39%) with a mean age of 58 years (SD 15.9, range 14–82 years). Patient's diagnoses are listed in Table 1.

**Table 1:** Diagnosis of patients who completed the Oxford hip score

**Diagnosis**	**Number of patients**	**% of patients**
Osteoarthritis	211	76%
Development hip dysplasia	33	12%
Avascular necrosis	15	5%
Juvenile chronic arthritis	5	2%
Rheumatoid arthritis	4	1%
Ankylosing spondylosis	3	1%
Hip fracture	3	1%
Psoriatic arthritis	2	1%

Forty-six (17%) patients annotated a total of 52 questions (Table 2). Five patients drew 16 arrows linking boxes, signalling that they felt they were unable to place themselves in a single category provided by the OHS. Question six, which asks "*During the past 4 weeks, for how long have you been able to walk before pain from your hip becomes severe (with or without a stick)?*" most frequently elicited annotation, whereas question eleven, which asks *"how much has pain from your hip interfered with your usual work (including housework)?" *was the only question that was not annotated by any of the patients. The annotations were broadly grouped into five main categories, each highlighting difficulties the patients experienced when completing the OHS (Table 3).

**Table 2:** Annotations on the Oxford hip score

**Question number**	**Annotations (n)**	**Total number of annotations**
1	Pain not constant in intensity (5), depends of medication (2)	7
2	Depends on part of body (2), due to other co-morbidities	3
3	More difficulty using public transport (4) depends on which side of the car, uses adapted taxi	6
4	Difficulty with and without an aid (4), depends whether it is socks, tights or stockings (2), Due to other co-morbidities	7
5	Due to other co-morbidities	1
6	Description of pain (5), difficulty with and without a stick/crutches (5), due to other co-morbidities (4), pain not constant in intensity, depends on medication (2)	17
7	Description of how stairs are climbed (4), uses stair lift	5
8	Pain not constant in intensity	1
9	In a wheelchair, reason for limp	2
10	Pain not constant in intensity, causes of pain	2
11	--------------------------------------------------------------------------------------	0
12	Causes of pain	1

**Table 3:** Categories of annotations made by patients on the Oxford hip score

**Category of annotation**	**Purpose of annotation**	**n (%) annotations**
Question clarity	To expand and explain answers	15 (29)
Measurement of pain	To explain nature of pain	12 (23)
Restrictive and irrelevant questions	To describe pain and alterations to activities, and comment on non applicable questions	12 (23)
Co-morbidities	To explain influence of co-morbidities on answer	7 (13)
Double-barrelled questions	To give two or more answers to a single question	6 (12)

## Discussion

The mean pre-operative OHS of 44.1 and post-operative score of 24.1 are similar to previous results [12,16,19], indicating that the sample in this study was representative of other lower limb orthopaedic patients. The pre-operative and the post-operative groups were purposively sampled as separate cohorts to avoid familiarly with the OHS, which could comprise the validity of the results. As the completion of the OHS was only introduced into this clinic in April 2004, the postal OHS completed by the post-operative patients was likely to be their initial contact with the questionnaire. Similarly, the patients attending the pre-admission assessment clinic should not have previously encountered the questionnaire. However, a limitation of the study was that patients may have previously completed the OHS for their GP or under the care of a different consultant, and this prior exposure to the OHS may have influenced the patient's responses.

This study has highlighted several pitfalls and limitations of the OHS, and of available disability measures in general. However, although the current study identified substantial areas of difficulty, analysing unprompted annotations has limitations. The results are confined to the difficulties encountered by individuals who were self-motivated to comment upon these problems. As a result of this methodology, conclusions are drawn from the responses of only 17% of the patients sampled. For the remaining 83% of patients, the OHS could have been adequate from their perspective or alternatively, they could have encountered problems, but not have documented them on paper as they were not instructed to do so. Therefore, further research needs to be undertaken, in which patient are explicitly encourage to comment upon any difficulties when completing the OHS, in order to assess the extent of it's applicability. Alternatively, qualitative interviews could be employed to explore the patient's perspective on the OHS in greater depth, although findings from qualitative work have raised similar areas of difficulty to those in the current study [23].

The five general themes of difficulties that emerged from the analysis of annotations is discussed in more detail below.

Seventeen percent of patients annotated answers they provided on the OHS, suggesting that the patients felt that the questions were inadequate to suitably express themselves. Five general themes emerged from the analysis of annotations and each thematic category is discussed in more detail below.

### Question clarity

The aspect of the OHS that appeared to cause the greatest difficulty for the patients, with 29% of annotations, was the lack of question clarity. Within this theme, the predominant area of uncertainty was whether the questions were enquiring about actual level of disability or the level of disability after accounting for the use of aids or specialised devices, such as long handled shoehorns or helping hands. When responding to question four, which asks respondents "*have you been able to put on a pair of socks, stocking or tights*?", a number of individuals answered accounting for the use of an aid, and other people gave two answers; one referring to the level of disability in performing the activity when using an aid and one when not using an aid. The same lack of clarity has resulted from this question previously [23]. Question six, which asks the respondent "*long have you been able to walk before pain from your hip becomes severe (with or without a stick)?*", acknowledges that many individuals need to use a walking stick. However, it is not specified in the question whether the patients should provide a response for actual or relative disability. Consequently, inconsistent results were obtained, with patients providing two answers i.e. distance walked with and without a walking stick. Therefore, the score becomes dependant on whether the respondent chooses to take account of the walking stick. These findings suggest that many respondents perceive the question as ambiguous. Further evidence for the lack of question clarity is based upon a large study of pre-operative patients, who most frequently omitted question six when completing the OHS [19].

In summary, it appears that the predominant area of ambiguity due to lack of question clarity on the OHS is whether patients should take into consideration the use of aids or specialised devices when responding to questions. Not taking consideration of the use of aids and devices, and indeed any assistance in activities, is a common oversight of many patient-centred measures of disability, such as the WOMAC [11]. Individuals who take into consideration the use of an aid when answering a question will appear less disabled than they are in reality. This lack of clarity could confound results, resulting in patients with the highest level of disability, who utilize specialised equipment in many activities, appearing to be the least disabled on paper. To enhance question clarity and gain consistent results it would appear advisable to specify to patients whether they should account for the use of aids or devices when responding to the question. However, modification of validated outcome measures can be fraught with problems [24], and therefore it may be more advisable to use an outcome tool that considers the modifying effect of aids and assistance on disability.

### Measurement of pain

Nearly a quarter of all the annotations provided an explanation of the nature of pain. Frequently patients commented that the intensity of pain can fluctuate greatly over four weeks and that the level of pain is heavily dependant on factors such as medication and activity. As a consequence, several patients felt they could not give an 'average' level of pain for the last four weeks. Therefore, a limitation of the OHS is that it attempts to categorise patients into a single category of pain when in fact pain, predominantly arthritic pain, is not static, but rather a dynamic entity. In a previous study, when interviewed about difficulties encountered when completing the OHS, individuals explained that they learned to ignore the pain, and that it could be masked by medication, and as a consequence struggled to complete the questions referring to pain [23]. Thus questions relating to 'average' pain appear inadequate to capture the experience of individuals with arthritic pain.

### Restrictive and irrelevant questions

Twenty three percent of annotations were descriptive or explanatory comments, supplementing the information recorded by the question. These annotations included descriptions of pain or how activities had to be modified as a consequence of disability, such as climbing stairs backwards, and the causes of pain. Furthermore, inadequate response categories resulted in 16 arrows being drawn between boxes, indicating patients were unable to place themselves into a single category. The original article on the OHS does not indicate how these responses should be scored [12]. Although it has recently been suggested that the highest score should be used, it may be argued that this is not a true reflection of the patient's answer and the clinician is introducing bias by selecting which answer to accept [24].

Expansion of answers was necessary for several patients to explain that, although they had answered the question, it was not applicable to them. Comments written in response to question seven, which asks *"have you been able to climb a flight of stairs?"*, suggest that climbing stairs is not applicable to everyone as some individuals have stair lifts installed or they live in a bungalow. In reply to question nine, *"have you been limping when walking because of your hip?"*, a respondent answered that they don't limp but explained this was a result of them being confined to a wheelchair. Although the questionnaire accounts for people that cannot drive by asking about difficulty travelling by public transport in question three, this question was not applicable to a patient who used an adapted taxi. The OHS appears to restrict individual's answers and fails to allow them to express themselves adequately, as well as including questions that are not relevant to all individuals.

### Co-morbidities

The OHS was designed as a site-specific outcome measure for orthopaedic evaluation, and as such, has been favoured over more generic outcome measures [16,21]. However, an underlying theme in the annotations was the difficulties that patients encountered when attempting to separate the disability and pain resulting from the affected hip from that arising from other co-morbidities. Contrary to Dawson and colleagues finding that the OHS is not influenced by co-morbidities [22], the effect size of the OHS has been found to be substantially smaller in patients with other mobility limiting conditions, compared with patients with unilateral hip osteoarthritis (OA), suggesting that other co-morbidities do influence the OHS [21]. The Oxford knee score, which has a comparable format to the OHS, produced similar results for patients with and without knee pain, in the presence of other co-morbidities, providing evidence that the questionnaire is not joint specific [25]. Patients with consistently high scores on the OHS have been found to suffer from multiple co-morbidities [18] and patients have verbalised that they find it difficult to separate pain from their hip from pain arising from other sites [23]. Therefore, co-morbidities appear to compromise the specificity of the OHS in evaluating disability resulting from hip symptoms, although joint specific questionnaires are designed to exclude the effects of co-morbidities.

In addition to the influence of diffuse co-morbidities, patients found it difficult to distinguish between pain originating from bilateral hips, highlighting a limitation of the OHS in considering only a single joint, which does not reflect the pattern of OA. In a sample of 500 OA patients, 53% of patients had more than one symptomatic joint [26]. Recently, this issue has been addressed by the modification of the OHS to ask about bilateral hip joints, although the success of this new design is questionable as 41% of the patients completed the OHS for the operated side only and 12% of patients did not discriminate between the two joints [24].

### Double-barrelled questions

During the validation process it is advisable to eliminate any double-barrelled questions [27], yet question three asks two questions in one: *"have you had any trouble getting in and out of a car or using public transport because of your hip?"*. Several patients answered the two parts of the question separately as it is common to use both modes of transport. Similarly, question four asks three questions in one: *"have you been able to put on a pair of socks, stocking or tights?"*. Again some patients answered this as three questions, with women often finding tights harder to put on than socks.

## Conclusion

The OHS is a useful short tool that is frequently utilised to assess the patient's perception of hip function, mobility and pain. It is quick both for the patient to complete and the clinician to score. Although the OHS is a widely used and validated patient-centred outcome tool, it appears that the OHS is not without problems, in concordance with previous findings [23]. It is unclear to patients whether the questions are asking about level of disability before or after accounting for the use of aids and devices. Individuals found it difficult to respond to questions about the severity of their symptoms due to the dynamic nature of pain and the use of medications to mask the pain. They also had difficulty separating other co-morbidities from the symptoms of the affected hip. Also double-barrelled questions caused confusion and not all questions on the OHS were relevant, or important, to the patient.

It could be argued that the difficulties patients experience with the OHS are due to the brevity of the scale, and could be reduced by the inclusion of additional questions. However, although there is little research on the problems experienced by patients while completing longer scales, such as the WOMAC [11] or HOOS [13], it appears that the limitations highlighted in the OHS could be applied to these longer questionnaires. The WOMAC does not account for the use of aids or devices, includes questions asking patients about their average pain level over the past 4 weeks, and has double barrelled questions such as "*what degree of difficulty do you have with getting in/out of bath/shower?"*. In addition, the WOMAC items have been found to be influenced by other co-morbidities, such as low back pain [28]. Previous research has found that the items on the WOMAC are unimportant, or irrelevant, to some individuals with OA [13]. This latter limitation is applicable to many validated patient-centred outcome measures. No single activity is important to all individuals, nor is the importance of being able to perform that activity necessarily stable over time [29]. Hence, an ideal would be to weight items of disability with the importance of performing that activity. This would allow non-applicable items to be rated as of no importance and thus not contribute to the score, producing a more individualised patient-centred outcome measure. Further work is underway to develop a personal impact of disability in osteoarthritis.

## Authors' contributions

VW was involved in the acquisition, analysis and interpretation of the data, and drafted the manuscript

IDL was involved in the conception of the study, revision of the manuscript and gave final approval of the version to be published

VJC was involved in the conception and design of the study and revision of the manuscript
